# Victims of Technology-Assisted Child Sexual Abuse: A Scoping Review

**DOI:** 10.1177/15248380231178754

**Published:** 2023-06-14

**Authors:** Katrin Chauviré-Geib, Jörg M. Fegert

**Affiliations:** 1 University of Ulm, Germany

**Keywords:** technology-assisted child sexual abuse, internet, victim, children’s experiences, abuse impact

## Abstract

Over the past two decades, technology-assisted child sexual abuse (TA-CSA) has become the focus of attention in politics, legislation, society, as well as research. However, the majority of literature and studies focus primarily on the offenders. This scoping review therefore aims to illustrate how victims of TA-CSA are represented in studies as primary participants. The databases Embase, PsychInfo, PSYNDEX, Cochrane Library, and Web of Science as well as reference lists were searched. Studies needed to be published between 2007 and 2021 and obtain data directly from and about victims to be included in this review. A total of 570 articles were identified from which 20 studies met inclusion criteria. The analysis showed that data can be obtained via different samples like adult and minor victims or other data such as legal documents or sexualized images. The studies researched different types of TA-CSA including exposure to pornographic material, online grooming leading to both online and offline sexual abuse, sexting and sexualized images, and the visual depiction of sexually explicit content. Consequences due to the abuse were of an emotional and psychological nature, medical or physical or impacted relationships, and the social environment. Even though the impact of the abuse on the victims appeared to be similar between different types of TA-CSA, much remains unknown. In order to gain further and more detailed insight into victims of TA-CSA, a universally accepted definition of TA-CSA as well as its different types and their distinctions needs to be established.

## Introduction

Recently, technology-assisted child sexual abuse (TA-CSA) is being increasingly focused on by the media as well as political stakeholders. The term TA-CSA encompasses all forms of child sexual abuse in which technology is used to initiate, escalate, as well as maintain the abuse (Hamilton-Giachritsis et al., 2017). Within three decades, institutions such as the European Police Office (EUOPOL) or the National Centre for Missing and Exploited Children have registered a continuous increase in the production and dissemination of TA-CSA (EUROPOL, 2020b). While the detection of TA-CSA had steadily increased over the years, a rapid spike was noted with the onset of the COVID-19 pandemic. In January 2016 INSAFE, a global network of helplines against TA-CSA, registered approximately 9,000 posts per day as being received by EU helplines whereas in March 2020 the number of posts had risen to 15,000 (EUROPOL, 2020a). Looking at a national example of administrative data, German law enforcement recorded 5,687 cases of distribution, purchase, possession, and production of material of TA-CSA in 2016, whereas the number of cases in 2021 had been multiplied by more than six times to 39,171 (Bundeskriminalamt, 2022).

The development of the web as well as technological advancements of modern information and communication technology in a short period is being accompanied by a new, digital crime scene. Around the turn of the century, Cooper (1998) already described how the web may be used for criminal activities and how this might be facilitated by the triple-A-engine: accessibility, affordability, and anonymity. National borders become less important whereas cybercrime increases its “popularity” due to simplified international communication of data. Platforms such as Boystown,^1^ which had been detected in April 2021, counted over 400,000 international members. Networking as well as dissemination of material of TA-CSA internationally is being facilitated by the advancing web and darknet (Ly et al., 2016). The continuous addition of a digital component in criminal offences suggests multiple crime scenes. Cases such as the Elysium platform^2^ or the German Staufen case^3^ depict both the range of the web as a crime scene and the growing linkage of hands-on (sexual abuse) with hands-off (possession and distribution of material of TA-CSA) offences. It is to no surprise that law enforcement agencies are increasing in work power as well as new technological prosecution methods which have become an essential part in the investigating process (Quayle, 2021). International as well as national task forces are formed, tasked with analyzing the rapidly increasing amount of material. Research on typologies of perpetrators as well as the correlation between paraphilic interests, the use of child sexual abuse material (CSAM), and the risk of offending have been increasingly addressed (Adebahr et al., 2021; Neutze et al., 2012; Wolak et al., 2011).

### Synonyms of Technology-Assisted Child Sexual Abuse

When dealing with TA-CSA, it is noticeable that a variety of terms are used synonymously, namely child pornography, CSAM, child sexual exploitation material, child sexual abuse images online, online facilitated child sexual abuse, among others. A common definition has not been established as all of the terms above cover a range of somewhat different, yet intersecting types of abuse. TA-CSA can therefore be used as a collective term to describe the use of technology in abuse. In most countries, child pornography is the legally correct term describing an offence of producing, possessing, and disseminating visual depiction of sexually explicit conduct involving minors (§ 184b Strafgesetzbuch [German penal code]; United States Criminal Code [18 USCS 2256]). As defined by the European Parliament, child pornography:

consists of images of child sexual abuse [ . . . ] by adults. It may also include images of children involved in sexually explicit conduct, or of their sexual organs [ . . . ] with or without the child’s knowledge. Furthermore, the concept of child pornography also covers realistic images of a child, where a child is engaged or depicted as being engaged in sexually explicit conduct for primarily sexual purposes. (European Parliament and of the Council of the European Union, 2011).

However, the term child pornography is criticized by experts. Pornography, in everyday language, refers to a consensual production of sexually explicit material of adults, a fictional depiction aiming to satisfy a sexual need. Its consumption is usually morally unproblematic. Based on age and development, children do not possess the understanding and required consent to partake in producing pornographic material or its consumption (Fegert, 2020) which is why using the same term signifies a terminological downplay of a serious offence. Hence, different terms describing TA-CSA are used among experts.

### Victims of Technology-Assisted Child Sexual Abuse

When talking about victims of TA-CSA the question arises of whom we are speaking about. Victims can roughly be subdivided into four categories when looking at the context of crime. The first way that TA-CSA victims can be conceptualized are sexually abused children of whose abuse some form of material had been created. Recorded sexual abuse might include material filmed for the abuser himself with no intention of dissemination or material aimed at exchanging with other recorded sexual abuse (Kriminalistisches Institut, 2019). The second concept contains children who are sexually abused via live streaming. This is a newer form of child sexual exploitation, benefitted by the COVID-19 pandemic, where offenders offer financial or other forms of gratuity to be able to watch the sexual abuse of children via a livestream and might instruct the course of the abuse (International Centre for Missing and Exploited Children, 2018; Napier & Teunissen, 2021). The abuses range from posing to sexual assault. (Tribolet-Hardy et al., 2020) The third way that victims can be conceptualized are children who are confronted with pornographic material against their will. A Swiss study about sexual abuse against children and adolescents (*n* = 6,749) reported that 2.1% of female and 2% of male participants had been confronted with a sexual act against their will. They had been forced or coerced to watch pornographic pictures, drawings, films, DVDs, or magazines (Schmid, 2012). The fourth concept of victims comprises children who sent pornographic material, in the form of a sext (e.g., picture, videos, voice notes, etc.) of themselves to a third party, whether supposedly willingly or coerced. A meta-analysis by Madigan et al. (2018), including 110,380 participants, identified a mean prevalence of 14.8% for sending and 27.4% for receiving sexts. In addition, the mean prevalence of forwarding sexting material without consent of the depicted person was 12.0%, whereas the prevalence of having sexting material forwarded without consent was 8.4%.

All of those listed above are victims of TA-CSA and yet, there are differences. Altogether, victims of TA-CSA are usually subjected to more than one form of abuse because in order to create digital material an actual sexual abuse needs to take place. Being exposed to more than one form of abuse can create diverse challenges for victims. As studies have shown, experiencing more than one form of abuse increases the likelihood of suffering from negative long-term effects compared to being subjected to a single form of abuse (Felitti et al., 2019; Hamilton-Giachritsis & Sleath, 2018; Witt, Brown, et al., 2019; Witt, Sachser, et al., 2019). Even though victims of sexual abuse do not show a specific set of symptoms and resilience exists more often than assumed (Domhardt et al., 2014; DuMont et al., 2007), the risk of negative consequences in terms of physical, psychological, and social impacts due to the abuse is accelerated as a result of the abuse (Teicher & Samson, 2013). Long-term consequences up into adulthood (Fergusson et al., 2013) can include anxiety and nervousness, posttraumatic stress disorder, avoidance strategies (Goldbeck, 2015), as well as physical health issues such as obesity and diabetes (Clemens et al., 2018). Recent studies suggest that negative symptoms of online sexual abuse are similar to those of offline sexual abuse (Joleby et al., 2020a).When a digital component is added in the abuse, literature suggests that feelings of powerlessness and helplessness increase as the existence and distribution of the material cannot be controlled by victims (Franke & Graf, 2016; Gewirtz-Meydan et al., 2018). Contrary to sexual abuse, being subjected to TA-CSA comes along with an ongoing victimization as online material can only be removed with great difficulty, if at all.

### The Current Study

As shown above, TA-CSA has been increasingly drawing attention but studies, discussions, and law-making mainly focus on perpetrators and the use of CSAM. Although knowledge on different typologies of perpetrators, how the use of material of TA-CSA is connected to sexual abuse offline, and the analysis of administrative data on cases of TA-CSA offer valuable insight into the protection of minors, the knowledge and perspective of victims of TA-CSA cannot be neglected. Instead, knowledge of victims outside of administrative data needs to be placed in the center of discussion as their expertise serves as a foundation in criminal and socio-political decision-making as well as therapeutic and preventive methods. It is therefore necessary to examine literature to provide an overview of studies where victims of TA-CSA are the primary participants so as to depict the scientific status quo and illustrate where future research is required. The following scoping review therefore focuses on literature concerning the question “How are victims of technology-assisted child sexual abuse material represented in studies as primary participants?”

## Method

A scoping review is a relatively new methodology whose application has rapidly increased over the past decade (Tricco et al., 2016). It was first characterized as examining the extent, range, and nature of research activity in order to draw conclusions about the overall state of research activity (Arksey & O’Malley, 2005). The current scoping review is based on the five-step methodical framework which was initially described by Aksey and O’Malley (2005) and further clarified and enhanced by Levac et al. (2010) as well as Pham et al. (2014). After (1) identifying the research question, (2) identifying relevant studies, (3) the study selection and (4) charting the data, (5) results are collated, summarized, and reported.

### Identifying Relevant Studies

To identify relevant studies, the following databases were searched between August and September 2022: Embase, PsychInfo, PSYNDEX, Cochrane Library, and Web of Science. To add to the database search, reference lists of the included studies and other articles that focused on TA-CSA but not the victims themselves were additionally hand-searched. The first author developed and implemented the search strategy with the assistance of a specialist librarian. Developing the search strategy included screening subject-specific articles beforehand as well as using the database thesaurus to determine synonyms of TA-CSA. As TA-CSA is described by different terms and victims are often partially included in studies, a broader search strategy was chosen to include as many articles as possible. An exemplary search strategy can be found in Supplemental Appendix A.

**Figure 1.**
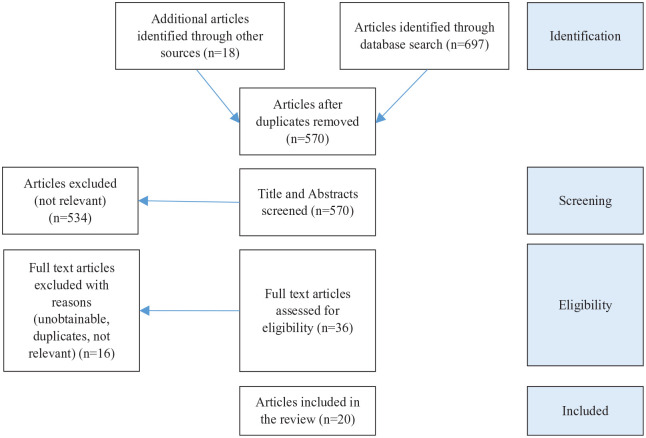
Preferred reporting items for systematic review.

The Preferred Reporting Items for Systematic Reviews and Meta-Analyses (PRISMA) according to Moher et al. (2009) were applied. Figure 1 illustrates the selection process from identification to inclusion according to the PRISMA guidelines.

### Inclusion Criteria

Only studies that obtained data directly from and about victims of TA-CSA were retrieved and qualified for inclusion. Victims were defined as children, adolescents, or adults who have been sexually abused as a minor (under the age of 18) and material of this sexual abuse has been produced. As TA-CSA is not yet homogeneously defined, the type of abuse was not limited. Furthermore, studies needed to be (1) written in English and (2) published between 2007 and 2021. The reasoning behind the initial date of articles to be included in the review lies in the onset of smartphone’s popularity after the demonstration of the first iPhone in 2007 and thus increasing technical possibilities of TA-CSA. Only primary or original studies were included.

Studies where (1) data on victims were included on a second-hand basis, for example professionals reporting on prevalence or evaluating perceived consequences for victims; (2) TA-CSA was not the main object of the study; and (3) reviews, commentaries, and conference abstracts were excluded.

### Study Selection

The review process was performed by the first author who conferred with the second author in cases of ambivalence. Six hundred ninety-seven articles resulted from the database search, another 18 articles were located through hand-searching reference lists adding to a total number of 715 articles. After excluding 145 duplicates, the remaining 570 titles and abstracts were screened creating initial categories in relation to the thematic focus of the articles. As a result, 534 articles were excluded as they were not relevant to the research question with over 50% of excluded articles focusing on the offender (*n* = 275). The 36 identified articles were then assessed for eligibility by means of a full text assessment. This led to the exclusion of another 16 articles. The excluded articles either could not be obtained (*n* = 1) or were not relevant to the research question (*n* = 15) as they did not focus directly on children as the affected victims of TA-CSA. Twenty studies met all inclusion criteria and were thusly included in the analysis (see Figure 1).

### Charting the Data

Following the reflexive thematic analysis of Braun and Clark (2019), descriptive data of the selected studies were initially summarized according to the JBI Reviewer’s Manual (The Joanna Briggs Institute, 2015) using a Microsoft Excel 2019 spreadsheet in order to get familiarized with the studies. This included author, year of publication, country of origin, title, focus of the study, study population, and sample size, design, victimization, as well as key findings that relate to the research question. Next, codes were identified by the first author which were then organized into key themes. When necessary, key themes were further specified or removed to accurately and authentically depict the existing data.

## Results

The aim of this paper is to examine how victims of TA-CSA are represented in studies. Following a descriptive overview of the included studies, the different types of TA-CSA and characteristics of the victims and the abuse are described as well as consequences and the impact on the victims. Twenty studies were relevant to the research question and met all inclusion criteria. The main characteristics of the included studies are presented in Table 1.

**Table 1.** Characteristics of the Included Studies.

Author (Year)	Origin	Focus	Victimization	Sample	Design
Canadian Centre for Child Protection (2017)	Canada	Experiences of victims, impact on victims	Recorded CSA	*N* = 150 adults, over the age of 18	Survey
ECPAT (2021)	Colombia	Victim’s recommendations for improving prevention and support services for children	CSEAO, CSAM, grooming for sexual purposes	*N* = 9 adults, aged 18–24	Qualitative
Gewirtz-Meydan et al. (2018)	United States	Experiences of victims	Child pornography	*N* = 133 adults, over the age of 18	Survey
Gewirtz-Meydan et al. (2019)	United States	Relationship between child pornography victimization and psychopathology in adulthood	Child pornography	*N* = 107 adults, aged between 18 and63	Survey
González-Ortega and Orgaz-Baz (2013)	Spain	Prevalence, extent, effects of minor’s exposure to online pornography	Exposure to pornographic material	*N* = 494 college students, mean age = 24.1 (*SD* = 5.6)	Survey
Hamilton-Giachritsis et al. (2017)	United Kingdom	Impact of online/offline CSA, improvement of response and professional’s perception	Grooming for sexual purposes, sexual solicitation	*N* = 246 young people, aged 15–20	Mixed-methods: Survey, qualitative
Joleby et al. (2020a)	Sweden	First-person perspectives, impact on victims	TA-CSA	*N* = 7 young people, aged 17–24	Qualitative
Joleby et al. (2020b)	Sweden	Children’s experiences and psychological health in written verdicts	OCSA	*N* = 98 legal cases, children aged 7–17	Qualitative
Jonsson and Svedin (2012)	Sweden	Characteristics of and possible consequences for child victims	Sexualized images	*N* = 11 teenagers, age NA	Qualitative
Jonsson et al. (2019)	Sweden	Adolescents’ experiences of online sexual contacts leading to online sexual abuse	OCSA	*N* = 5,715 high school students, mean age = 17.97 (*SD* = 0.63)	Survey
Katz (2013)	Israel	Narratives of alleged victims of child sexual abuse through the Internet	Grooming, CSA	*N* = 20 legal cases, children aged 11–14	Qualitative
Mitchell et al. (2011)	United States	National estimates of youth involved in sexting, characteristics of victims and material	Sexting	*N* = 1,560 minors, aged 10–17	Survey
Quayle and Jones (2011)	United Kingdom	Characteristics of children depicted in child abuse images	Sexualized images	*N* = 24,500 sexualized images, age NA	Descriptive
Quayle et al. (2012)	Sweden, United Kingdom, Germany, Italy, Denmark, Russia	Experiences of young people involved in online grooming	Grooming, CSA	*N* = 27 minors, aged 11–17	Qualitative
Quayle et al. (2018)	United Kingdom	Characteristics of children depicted in sexualized images	Sexualized images	*N* = 687 sexualized images, mean age = 11.1 (*SD* = 4.29)	Descriptive
Sabina et al. (2008)	United States	Prevalence and experiences of exposure to online pornography as a minor	Exposure to pornographic material	*N* = 563 college students, median age = 19	Survey
Salter and Whitten (2021)	Australia	Characteristics of children depicted in sexualized images; comparison between pre-internet sexual abuse material and contemporary dataset	Sexualized images and other digital media	*N* = 35,565 sexualized images, aged 0–17	Descriptive
Soo et al. (2012)	Estonia	Association between exposure and behavioral, psychological, and demographic characteristics as well as social mediation of internet use	Exposure to pornographic material	*N* = 780 minors, aged 11–16	Mixed-methods: Survey, qualitative
Whittle et al. (2013)	United Kingdom	Impact on victims of online grooming leading to online/offline sexual abuse	Grooming, CSA	*N*=8 minors, aged 12–14	Qualitative
Wolak et al. (2018)	United States	Analysis of sextortion as well as differences between minors and young adults	Sextortion	*N* = 1385 adults, aged 18–25	Survey

*Note*. CSA = child sexual abuse; CSEAO = child sexual exploitation and abuse online; CSAM = child sexual abuse material; TA-CSA = technology-assisted child sexual abuse; OCSA = online child sexual abuse; NA = not available; *SD* = standard deviation.

### Descriptive Outcomes

Databases were searched for articles that had been published between the years of 2007 and 2021. Relevant studies could be identified in 9 years out of this 15-year timespan at a rate of 1 to 3 per year: 2008 (*n* = 1), 2011 (*n* = 2), 2012 (*n* = 3), 2013 (*n* = 3), 2017 (*n* = 3), 2018 (*n* = 2), 2019 (*n* = 1), 2020 (*n* = 2), 2021 (*n* = 2). Most studies originated from Europe (*n* = 10), followed by studies from North and South America (*n* = 7). Furthermore, one study originated from Australia, one from Israel and another was a mixed study of European and Russian researchers. Different study designs were used, such as online surveys or questionnaires (*n* = 7), interviews (*n* = 5), descriptive analysis of sexualized images (*n* = 3), qualitative analysis of court files (*n* = 2), a mixed-method approach including questionnaires and interviews (*n* = 2), as well as a telephone survey (*n* = 1). Fourteen studies underwent a peer-review process in order to be published. Of the remaining six studies, three were published as a project report.

The majority of the studies (*n* = 7) included adults reporting retrospectively on TA-CSA, from which two studies were conducted with college students (González Ortega & Orgaz Baz, 2013; Sabina et al., 2008) focusing exclusively on exposure to pornographic material online as a minor. In six studies, the main participants were minors, their age ranging between 10 and 17 (Jonsson & Svedin, 2012; Jonsson et al., 2019; Mitchell et al., 2011; Quayle et al., 2012; Soo et al., 2012; Whittle et al., 2013). Two studies included both minor and young adult participants being described as young people aged 15 to 20 (Hamilton-Giachritsis et al., 2017) and 17 to 24 (Joleby et al., 2020a). The remaining five studies analyzed either legal documents (Joleby et al., 2020a; Katz, 2013) or sexualized images (Quayle & Jones, 2011; Quayle et al., 2018; Salter & Whitten, 2021), where the age of the affected child, if known, ranged between birth and up to 18 years of age. In total, data was directly obtained from 11,195 victims across all studies, ranging from *n* = 7 (Joleby et al., 2020a) to *n* = 5,717 (Jonsson et al., 2019), whereas studies analyzing other data combined an overall sample of *n* = 60,870.

To identify and describe the impact of the abuse was the purpose in eight studies, whereas it was not always the sole objective: in four studies, a specific abuse was researched as well as its impact on the victims. Quayle et al. (2012) and Whittle et al. (2013) explored online grooming leading to online and offline sexual abuse and its impact on the child. Jonsson and Svedin (2012) as well as González Ortega and Orgaz-Baz (2013) focused on minors’ exposure to pornographic material online and its impact on the children. Quayle and Jones (2011), Quayle et al. (2018), and Salter and Whitten (2021) aimed to describe the children within the image using sexualized images as their data. The focus of two studies, in which legal documents were analyzed, was to show and characterize the presence of children in the legal process, particularly in the documentation of legal documents (Joleby et al., 2020a; Katz, 2013). A study of victims’ perspectives in Colombia (ECPAT International and Fundación Renacer, 2021) aimed at working out improvements in prevention as well as support services for affected children.

## Different Types of Technology-Assisted Child Sexual Abuse

Most of the included manuscripts tried to give a definition, often describing aspects of the abuse. When describing the same type of abuse, the same definition was rarely used more than once or twice even though the described aspects overlapped. When looking at the types of TA-CSA in the included studies, a subsumption was possible.

Sabina et al. (2008), Soo et al. (2012), and González Ortega and Orgaz-Baz (2013) examined the *exposure to pornographic material as a minor*. Soo et al. (2012) described exposure to pornographic material more generally as being exposed to online sexual messages and harassment including receiving or seeing sexual messages, that is in words, pictures, or videos. Sabina et al. (2008) and González Ortega and Orgaz-Baz (2013) determined exposure similarly as having seen online pornography as a minor and included more detail as to what that entails: “images of paraphilic or criminal sexual activity including child pornography and sexual violence” (Sabina et al., 2008, p. 691), being sought out explicitly or being exposed to involuntarily as well as:

images of naked people, naked people showing genitals in explicit poses or people having sex, images of deviant or criminal sexual activity, sexual bondage, sexual activity between people and animals, sexual activity involving urine or feces, child pornography and/or rape or sexual violence. (González Ortega & Orgaz Baz, 2013)

Quayle et al. (2012), Katz (2013), and Whittle et al. (2013) focused on *online grooming leading to sexual abuse both online and offline*. The two latter used the same definition of online grooming according to Craven et al. (2006) who defined it as a process of preparing a child, significant adults and the environment for the abuse of this child. Furthermore, grooming involves gaining access to the child as well as its compliance. To avoid disclosure, the child’s secrecy needs to be maintained. Quayle et al. (2012) were not as clear in describing online grooming but referred to it more from the victim’s perspective as a liking of the online contact as well as the person met online leading to being groomed or lured into receiving solicitations and requests for sexual images or sexual contact.

Another type of TA-CSA could be subsumed under the broad term of *sexualized images on the internet*. Different phenomena such as *sexting*, *CSAM*, and *sextortion* were researched but often overlapped in the following studies blurring the difference between the types. Research over the past years has increasingly focused on the exchange of sexual texts or pictures between adolescents as part of their development, also known as *sexting*. That by itself is not seen as problematic as long as it is voluntary. An explorative curiosity of sending and receiving sexts can however transition into a problematic behavior where an explicit distinction between voluntary and involuntary actions is necessary. *Sexting* as a type of TA-CSA was explicitly only studied by Mitchell et al. (2011). Several other studies mentioned sexting as part of their research (Hamilton-Giachritsis et al., 2017; Joleby et al., 2020a; Wolak et al., 2018) or categorized the phenomena of sexting as part of sexualized images on the internet (Jonsson & Svedin, 2012; Quayle & Jones, 2011). In Quayle et al. (2018), sexualized images were defined as images online depicting children in sexual situations, while sexting comprised posting or sending sexually suggestive images such as nude or semi-nude. It was differentiated between self-taken images versus images taken by others. Salter and Whitten (2021, p. 1120) used the description of CSAM defining it as “sexually exploitative images and videos of minors as well as texts, drawing, and computer simulations of child sexual abuse.” Jonsson and Svedin (2012) characterized CSAM as under-aged children being depicted in sexual images online. They outlined the concept of CSAM being often equated with sexually abusive images or child pornography highlighting the need to differentiate between the images. As proposed in their paper, sexualized images can be distinguished between self or in consensus produced material and material being produced during a sexual abuse. Later on, the distribution of the material can be divided into self-distributed (as might be the case in sexting where self-produced pictures are deliberately sent to someone), distributed by another with or without permission and lastly distributed by the perpetrator after the sexual abuse. The term *sextortion* was only used by Wolak et al. (2018) as they aimed for a better understanding. They described it as a phenomenon in which a perpetrator threatens to expose sexual images in order to coerce victims into providing additional sexual material, engaging in sexual acts or consenting to other demands (Wolak et al., 2018). The authors highlight that terms such as sexting, non-consensual sharing of sexual images or revenge pornography may include sextortion but should not be used synonymously. Sextortion itself is defined by the threat to expose already existing material in order to coerce a certain behavior.

The *visual depiction of sexually explicit conduct* was another type of TA-CSA that was studied by three research groups (Canadian Centre for Child Protection, 2017; Gewirtz-Meydan et al., 2019; Gewirtz-Meydan et al., 2018). Being described as child sexual abuse that had been recorded, the visual depiction of sexually explicit conduct might be the “typical” type of TA-CSA including intercourse, masturbation, and exhibition of the genitals or pubic area (Gewirtz-Meydan et al., 2018). Gewirtz-Meydan et al. (2019; 2018) also used the controversial term of child pornography stating that even though the term is intensely discussed and disliked by many professionals it is being used due to its legal understanding and application.

The remaining five studies did not examine one type of TA-CSA exclusively but rather used wider definitions that included the types described above. For instance, ECPAT (2021) summarized acts such as the production, possession, and dissemination of CSAM; online grooming for sexual purposes; live streaming of child sexual abuse; and other practices such as unwanted exposure under the term of child sexual exploitation and abuse online. Jonsson et al. (2019) focused on online sexual contacts leading to online sexual abuse. To measure sexual abuse, six aspects were specifically listed: “someone flashed in front of you, touched your genitals, you masturbated someone, oral, vaginal or anal penetration” (Jonsson et al., 2019, p. 3). Hamilton-Giachritsis et al. (2017) used the term of technology-assisted sexual abuse synonymously to online child sexual abuse characterizing it as a range of activity including offline child sexual abuse shared with and viewed by others via technology (for example live streaming of sexual abuse via a webcam), offline sexual blackmail, online grooming, sexting, etcetera. The term *technology-assisted child sexual abuse* was applied in the study by Joleby et al. (2020b) where it included sexual conversations, sexual posing, masturbation, as well as penetration. The particular characteristic they focused on was the active participation of the victim in the abuse. As a result of the perpetrator’s encouragement, victims seemingly willingly partook in the abuse. With this, a clear differentiation to other forms such as online dissemination of abuse pictures, online grooming, or sextortion was made. Another article by Joleby et al. (2020a) focused on the victim’s seemingly active participation in the abuse as well but addressed it as online child sexual abuse. This abuse can include producing nude or semi-nude pictures or performing sexual acts live in front of a webcam as well as leading to an offline meeting with the perpetrator which included contact sexual abuse.

### Characteristics of Victims and the Abuse

Most studies gave a description of the victims characterizing them as well as the abuse. Following the subsumption of described types of abuse into categories, victims and the abuse can be described as followed.

In the studies on *exposure to pornographic material*, the prevalence in regard to the quantity of the sample ranged between 19 and 72% (González Ortega & Orgaz Baz, 2013; Sabina et al., 2008; Soo et al., 2012). However, that range might be explained by the sample itself as a higher prevalence was discerned by a retrospective data collection of adults (González Ortega & Orgaz Baz, 2013; Sabina et al., 2008) compared to surveying minors (Soo et al., 2012). The age at first exposure was fairly similar and described between the ages of 14 to 17 (Sabina et al., 2008) or as the mean age of 15 (González Ortega & Orgaz Baz, 2013). The similarity between the studies of Sabina et al. (2008) and Gonzáles Ortega and Orgaz-Baz (2013) can be explained by the fact that the latter based parts of their survey on the study of the first. All three studies detected a significantly higher involuntary exposure for girls as well as girls being more often targeted with sexual messages and more likely disturbed by it.

In regard to *online grooming*, both online and offline assault was a direct result of online communication. Katz (2013) described a linear process of grooming which could partially be found in the studies by Quayle et al. (2012) and Whittle et al. (2013): after (1) an approach through the Internet, (2) online communication and building of a rapport took place. Following (3) the perpetrator usually asked for a phone number or meeting offline where (4) the sexual abuse took place. The grooming process could take place during a few months (Katz, 2013) or between 10 days up to 18 months (Whittle et al., 2013). The form of abuse included both online and offline sexual abuse such as vaginal, oral, or anal penetrative sexual abuse (Katz, 2013; Quayle et al., 2012; Whittle et al., 2013) and sending naked photos (Katz, 2013; Whittle et al., 2013). All three studies determined an active pursuit of online contacts by the ultimate victims, sometimes directly looking for communication surrounding sexual matters. It was described as a search online for a way of meeting one’s needs including looking for a trusted person to talk to or satisfying curiosity. Another motivation to look for someone to communicate online with was described by Quayle et al. (2012) as a wish and need for control which was often synonymously used with feeling safe and self-confident in a new-found relationship online. The age at the time of the assault was placed between 11 and 17 by Quayle et al. (2012) and 11 to 14 by Katz (2013), whereas Whittle et al. (2013) exclusively studied victims that were groomed online between the ages of 12 to 14. Over 75% of the victims in all three studies were female which is in line with a risk factor of receiving sexual requests online being female (Mitchell et al., 2007). The victims in Katz’s (2013) study did not know the perpetrator prior to the onset of the abusive situation. At some point during the contact, all female participants in Whittle et al.’s (2013) study claimed the abuser to “be a boyfriend.”

The studies that analyzed *sexualized images* all reported on the majority of children in the pictures being female (Quayle & Jones, 2011; Quayle et al., 2018) though the age distribution differed. During Quayle and Jones (2011) analysis it became clear that that the majority of the depicted boys in the images tended to be prepubescent (73%) whereas depicted girls were almost equally divided between being prepubescent (51.4%) and pubescent (47.9%). Another sample analysis by Quayle et al. (2018) showed no age difference between genders. Salter and Whitten (2021) compared a pre-internet sample of sexualized images with a contemporary sample which indicated an age shift for girls being younger in more recent images. When surveying young internet users, Mitchell et al. (2011) reported that the prevalence differed depending on the definition of sexting, in particular which activities were implied. They highlighted that the percentage of young internet users who fall under a definition that can be seen as legally critical is very low, no more than 1% of their sample. The content of the sexualized images described by Quayle et al. (2018) included mostly nudity or erotic posing with no sexual activity. Comparing content of sexualized images between a pre-internet and a contemporary sample, the increased severity of abuse over the years was noted. This was indicated by the increased presence of an adult abuser in the images as well as the presence of an animal in the abuse (Salter & Whitten, 2021). Although many images were taken in a relationship of some sort, the origin appeared to be involuntary on a subconscious level. Even though victims sent images voluntarily, they reported feeling pressured to provide in order to maintain the relationship (Wolak et al., 2018). Similar findings resulted in the analysis of Quayle et al. (2018) where two-thirds of self-taken images were classified as coercive. Resembling the motivation of looking for contact online resulting in online grooming, victims reported a need for attention as well as sexual curiosity as their motivation for taking part in the exchange of sexualized images (Jonsson & Svedin, 2012).

The abuse of children of whom *visual depictions of sexually explicit conduct* had been produced began at a young age. In the study by the Canadian Centre for Child Protection (2017), 56% of victims reported that the abuse started between birth and 4 years of age. In the study by Gewirtz-Meydan et al. (2018, 2019) the mean age when the images were first created was 6. Prominent in all three studies is the duration of the abuse, 80% continuing over a year as well as into adulthood, meaning that they had partially been abused for over 10 years. As the perpetrators were often part of their direct family members living under the same roof, the duration of the abuse without being discovered by a third party seems plausible. In the same study, 68% of the victims reported additionally that the imagery of the abuse was not discovered at the same time as the hands-on abuse but later on. Victims of *technology-assisted child sexual abuse* showed similar characteristics including age at time of the abuse being between 7 and 17 and mainly being female (Joleby et al., 2020a, 2020b) as well as the abuse ranging from a single incident up to several years (Hamilton-Giachritsis et al., 2017; Joleby et al., 2020a, 2020b). The documented abuse included sexual posing, masturbation, penetration, as well as the involvement of another person or animals; the majority of the victims did not know the perpetrator prior to the onset of the abusive situation (Joleby et al., 2020b). The study of Jonsson et al. (2019) furthermore identified that victims of online sexual abuse had been significantly more often exposed to different forms of abuse such as penetrative sexual abuse offline and physical abuse while growing up as well as having a significantly poorer relationship with their parents.

### Consequences and Impact of the Abuse

Most included studies generally addressed one or more aspects of the impact of the abuse and its consequences. The following three categories can be identified throughout the studies: (1) emotional responses and psychological impact, (2) medical and physical impact, and (3) the impact on relationships and the social environment.

Regarding victims’ emotional responses as well as the psychological impact of the abuse, some research groups noted that these were not exclusively negative but positive reactions could be identified as well. Even though it might lead to other, more negative reactions later on, sexual excitement and sexual curiosity were frequently named as positive reactions by victims concerning the abuse (González Ortega & Orgaz Baz, 2013; Jonsson & Svedin, 2012; Katz, 2013; Sabina et al., 2008). However, the most immediate emotional responses to the abuse were negative. Ten of the included studies identified feelings of embarrassment, guilt, and shame as the most immediate reaction followed by disgust, shock, fear, and anxiety (Canadian Centre for Child Protection, 2017; Gewirtz-Meydan et al., 2018; González Ortega & Orgaz Baz, 2013; Hamilton-Giachritsis et al., 2017; Joleby et al., 2020a; Jonsson & Svedin, 2012; Katz, 2013; Mitchell et al., 2011; Quayle et al., 2012; Sabina et al., 2008; Salter & Whitten, 2021; Whittle et al., 2013). Jonsson and Svedin (2012), Whittle et al. (2013), and Joleby et al. (2020a) furthermore detected low self-esteem, aggression, sexualized behavior, and self-harming as a result of the first emotional response. Victims often blamed themselves for actively participating in producing the images and that they were not able to stop the abuse (Hamilton-Giachritsis et al., 2017; Joleby et al., 2020b; Katz, 2013). Additionally, professionals noted that victims of TA-CSA were more often blamed as having actively participated in the abuse than victims of offline sexual abuse (Hamilton-Giachritsis et al., 2017). Gewirtz-Meydan et al. (2018) observed that emotional responses of guilt and embarrassment at and shortly after the crime were significantly associated with increased psychopathology. During interviews by Joleby et al. (2020b), it became obvious that the abuse had a long-lasting impact on the victims as their self-perception had changed radically. Similar findings were identified by Katz (2013) and Sabina et al. (2008) where victims described themselves as prostitutes, felt unattractive, inadequate, or stupid after the abuse. At times, being a victim of TA-CSA resulted in changed behavior and thought patterns such as being less eager to seek sexual experiences or having unwanted thoughts about the material at random times (Sabina et al., 2008). Individual studies furthermore focused on similarities and differences between TA-CSA and sexual abuse which occurred exclusively offline. Jonsson and Svedin (2012) as well as Hamilton-Giachritsis et al. (2017) emphasized that there were no different symptoms between victims of offline and online abuse. Based on their findings, they objected the presumption that TA-CSA might be less impactful than offline sexual abuse.

As described by Wolak et al. (2018), 29% of the victims went to see a mental health or medical practitioner as a result of the sextortion incident. The only other physical impact of an abuse that was reported in the studies was sleeplessness (Canadian Centre for Child Protection, 2017; Joleby et al., 2020b).

A factor which stood out significantly was that victims were fearful of the reactions from people they knew once the abuse was exposed. Namely, victims’ perceived willingness to participate in the abuse or being recognized caused distress (Canadian Centre for Child Protection, 2017; Gewirtz-Meydan et al., 2018). Victims also feared that others might consider them to be at fault for the material being created which then led to further anxiety (Gewirtz-Meydan et al., 2018; Joleby et al., 2020a). Another consequence of the abuse was impaired relationships (Joleby et al., 2020a). This was described as a lack of parental trust (Whittle et al., 2013) or more generally strained relationships with family members (Canadian Centre for Child Protection, 2017) as well as losing relationships with family and friends (Wolak et al., 2018). In addition, educational and professional achievement were severely limited by the abuse as victims reported having school-related problems right up to having to leave or change schools as well as difficulties gaining and maintaining employment (Canadian Centre for Child Protection, 2017; Wolak et al., 2018). The critical findings of this review are summarized in Table 2.

**Table 2.** Critical Findings.

TA-CSA covers a range of different types including exposure to pornographic material as a minor, online grooming leading to sexual abuse online and/or offline, sexualized images, and the visual depiction of sexually explicit conduct.
Victims of TA-CSA are represented among all age ranges and gender, whereas the majority were female.
The impact of the abuse on the victims were mainly negative and can be categorised in emotional responses and psychological impact, medical and physical impact, and the impact on relationships and the social environment.

*Note*. TA-CSA = technology-assisted child sexual abuse.

## Discussion

This study aimed to illustrate how victims of technology-assisted child sexual abuse (TA-CSA) are represented as primary participants in studies. Firstly, while general research on TA-CSA has increased over the past years, studies focusing directly on victims are still rare. The studies included in this review showed that victims of TA-CSA are exposed to different types of abuse including exposure to pornographic material, online grooming leading to both online and offline sexual abuse, sexting, and sexualized images and the visual depiction of sexually explicit content. However, a quarter of the included studies of this scoping review did not give a specific definition or association to one of the named types of abuse but rather used a broad terminology. There is an obvious need for defining TA-CSA in general but more importantly to distinguish the types of abuse of TA-CSA. First of all, future research should create a universal definition of and language relating to TA-CSA and a clear distinction of its types. Distinct definitions allow for more specific research questions and prevent wasting resources. Consequently, more in-depth understanding of the individual type of TA-CSA itself, its causes and consequences for victims is gained which can then be used for practical application. Future studies need to build stronger evidence of the different types by using distinct definitions as well as different study designs on the same type so that a body of knowledge can be built over time. Definitions should therefore be empirically tested and implemented as well as operationalized in practice. Only if sufficient basic knowledge about the individual types is obtained, a comparison between different types is possible. Already, the different studies indicate that there are similarities of the impact and consequences victims experience during different types of abuse.

Secondly, this scoping review revealed that different samples can be used to gain insight into victims of TA-CSA including adults, minors, and other data such as legal documents or sexualized images (see Table 1). Each type of data acquisition has its justification, and researchers need to consider the respective advantages and disadvantages of the sample. Valuable insight can be gained into the abuse from a victim’s perspective. Accurate data on the actual extent of child victimization are crucial in developing prevention strategies and policy initiatives (Finkelhor et al., 2016; Joleby et al., 2020a). While offering valuable insight into the abuse, surveying minors also presents challenges as, for example, data needs to be viewed within the context of the minor’s developmental stage. Smaller children might not be able to explicitly describe a past situation or might use wording that can be understood differently than intended. There are also differences in the ability to recall a certain situation depending on age (Fivush, 2011) as well as the ability to comprehend questions and answer accordingly (Poole, 2016). A study by Walsh et al. (2018) investigated the willingness of minor victims portrayed in sexual abuse images and their parents to partake in studies concerning their abuse. They identified a relatively high percentage of consent (Walsh et al., 2018). However, some of the included studies in this review that interviewed minors of TA-CSA reported difficulties in recruiting a satisfactory number of participants. In comparison, access to adult victims as primary participants is usually easier. If data is collected from a representative sample, the said data can be used to ascertain the prevalence of certain types of TA-CSA, for example. Nevertheless, a retrospective collection of data does not allow for causality. Moreover, the main criticism of retrospective surveys lies with retrospective accounts being prone to recall bias (Hardt & Rutter, 2004) as participants may not remember situations accurately especially if the experience had been emotionally straining. Analyzing other data such as legal documents or images can be done independent of time and people, which is an advantage. Especially when cases are not frequent, data analysis can generate viable results from small samples, for example due to standardized forms in legal documents. Accessing court records or international data bases such as the International Child Sexual Exploitation Image Database (Quayle et al., 2018) might be complicated though due to complex requirements in order to gain access. Data analysis also does not conclude in representative results as well as it often consists of complex analysis, including developing and validating coding or programming an Artificial Intelligence to search for marks.

Thirdly, there seem to be similarities in the reported impact of different types of TA-CSA. In the studies the negative psychological effects on the victims of the abuse were predominantly reported on. For many victims there appear to be heightened feelings of shame and guilt. Also, as victims feared that outsiders would be under the assumption that they had voluntarily participated in the production of the material, they questioned whether they were victims at all. Feelings of shame can be a response to trauma as CSA itself is an act of violence in which the victim is not able to maintain personal boundaries (Frazier, 2000). Studies focusing on a correlation between trauma and CSA report no gender differences regarding the experienced post-abuse shame (Dorahy & Clearwater, 2012; Rahm et al., 2006), but suggests that adolescents who experienced sexual abuse-induced shame are more vulnerable to mental health problems (Ellenbogen et al., 2018). Findings from the relationship between CSA and shame can only be transferred to TA-CSA in a rudimentary manner though. The digital component of TA-CSA is likely to bring additional challenges. Therefore, future studies need to focus explicitly on TA-CSA and shame as well as exploring possible gender differences. Furthermore, a common negative impact of TA-CSA is that the perceived reactions of third parties causes additional distress. Disclosing sexual abuse is a necessary step in processing a possible traumatic experience. Delayed disclosures can have detrimental psychological effects, whereas positive disclosure promotes a healthy adjustment (Hall et al., 2023). Hence, the reactions received on disclosure can either positively or negatively shape the further process. Professionals have a responsibility when TA-CSA is disclosed to them as their reaction can either directly minimize or maximize the impact of the abuse (Whittle et al., 2013).

Even though the included studies originated from various countries, they were predominantly conducted in Western countries. Some studies, mainly those in the United States, included ethnicity when describing the characteristics of the sample but did not further elaborate possible differences in their findings. Neither ethnical nor cultural factors that might have influenced the victims’ willingness to open up about their experiences or their perception of the abuse were discussed. Soo et al. (2012) highlighted that their findings regarding differences between Estonian-speaking and Russian-speaking children showed that future research must address ethnicities in more detail. Only one other European study that obtained data directly from victims mentioned that their findings could not be generalized due to the sample being disproportionately white and female (Hamilton-Giachritsis et al., 2017). Studies that analyzed sexualized images highlighted that white as well as female victims were disproportionally represented (Quayle et al., 2018; Salter & Whitten, 2021). In order to discuss similarities and differences caused by culture and ethnicity, future studies and sampling need to reflect a more diverse nature and include ethnic minorities and male victims.

Some limitations of this scoping review need to be addressed. The authors are aware that the current scoping review probably does not include all studies that portray victims of TA-CSA due to imprecise definitions. For example, many studies that address the topic of sexting mainly focus on the phenomenon itself: how it developed over the past years or how it is influencing development in regards to social interactions as well as mental stimuli. The criminogenic aspect of sexting, however, is mentioned only marginally or not being clearly described and therefore not associated with TA-CSA. Although the search strategy was designed to be as thorough as possible using various synonyms of TA-CSA, there is no guarantee that all relevant studies were found in the data bases and reference lists. Secondly, varying definitions of the term “child” proved to be a challenge. In most countries, a minor is a person under the age of 18. Ireland and Luxembourg, for example, define children to be under 18 with no further distinction. However, some countries, such as Germany and Bulgaria, distinguish further, sometimes even with different legal regulations, between adolescents aged between 14 and 18 and children aged below 14. Thirdly, a limitation of the current study was the sample sizes of some of the included studies. When researching victims, especially minors at the time of the study, the access to participants is difficult due to the sensitivity of the topic as well as legal reasons as consent is needed from a legal guardian. Small sample sizes do suggest findings but make it difficult to detect a significant effect. While similar impacts of the abuse such as feelings of shame, guilt, and embarrassment were found in most studies, one still needs to be careful in generalizing the findings. Therefore, the findings of the current scoping review cannot be generalized either.

## Conclusion and Implications for Practice and Policy

The current scoping review finds that studies focusing exclusively on or collecting data directly from victims of TA-CSA are still rare. With the rapid increase of TA-CSA related offenses, knowledge about its victims needs to increase. Analyzing the different types of TA-CSA is essential to improve the understanding of the impact of the abuse, possible gender differences, as well as cultural factors. As presented in this scoping review, victims of TA-CSA can be faced with severe consequences. Therefore, preventive and intervention programs need to address their specific needs. Among professionals, an awareness needs to be created of TA-CSA in general as well as its different types so that disclosure is made easier for victims. When the crime, its legal implications, or offenders are studied, the victims themselves are subjected to an objectification. Insight gained directly from victims plays an important role in preventive and therapeutic methods and need to be considered further when discussing legal alterations. For summarized applications for research, practice, and policy, see Table 3.

**Table 3.** Implications for Practice, Policy, and Research.

Practice	Among professionals, an awareness of different types of TA-CSA needs to be further established. Adequate responses when learning about TA-CSA is required in order to counteract possible negative impacts on victims.
Policy	When drafting new bills or altering existing law, victims’ voices need to be considered.
Research	Different types of TA-CSA have been addressed across the identified studies using different yet overlapping definitions. Further research needs to create universal definitions and a clear language about TA-CSA and tis types. Various research designs should be applied in order to build a body of knowledge. Only 20 studies between 2007 and 2021 addressed victims of TA-CSA as primary participants. It is necessary to further research victims directly in order to gain insight into protective and risk factors, impact and consequences of the abuse, as well as differences and similarities of TA-CSA to experiencing child sexual abuse without a digital component.

*Note*. TA-CSA = technology-assisted child sexual abuse.

## Supplemental Material

sj-docx-1-tva-10.1177_15248380231178754 – Supplemental material for Victims of Technology-Assisted Child Sexual Abuse: A Scoping Review Supplemental material, sj-docx-1-tva-10.1177_15248380231178754 for Victims of Technology-Assisted Child Sexual Abuse: A Scoping Review by Katrin Chauviré-Geib and Jörg M. Fegert in Trauma, Violence, & Abuse

## Notes

1. Boystown was the world’s largest platform for child pornography on the Darknet until it was discovered in 2021. At the time of exposure, there were over one million forum posts, some of them depicting severe child sexual abuse.

2. Elysium was a Darknet website that distributed child pornography in the form of images and videos. In 2017, it was shut down by investigative authorities.

3. The Staufen case is a case of sexual abuse in Germany that was detected in 2017. A boy was brutally raped over a period of 2 years by his biological mother and her partner, a previously convicted sex offender who had been banned from contact with children. The victim was also sold to other male perpetrators in the Darknet and filmed during the abuse.
