# Supplementary material for: Victims of Technology-Assisted Child Sexual Abuse: A Scoping Review
Source: Trauma Violence Abuse. 2023 Jun 14;25(2):1335–48. doi: 10.1177/15248380231178754 (PMC10913305; doi:10.1177/15248380231178754)
Supplement: sj-docx-1-tva-10.1177_15248380231178754 – Supplemental material for Victims of Technology-Assisted Child Sexual Abuse: A Scoping Review [file sj-docx-1-tva-10.1177_15248380231178754.docx]

**Appendix A**

## Exemplary search strategy

Search carried out in EMBASE (OVID) for the period 1980 to 2021 Week 52. Date of search 28/09/2022

| ***Search strategy*** | |
| --- | --- |
| 1 | child sexual abuse material |
| 2 | child sexual abuse image* |
| 3 | child sexual abuse image* online |
| 4 | online child sexual exploitation |
| 5 | child sexual exploitation material |
| 6 | Child sexual exploitation image* |
| 7 | child sexual abuse and exploitation image* |
| 8 | online facilitated child sexual abuse |
| 9 | child pornography |
| 10 | child pornographic exploitation |
| 11 | child abuse material |
| 12 | child exploitation material |
| 13 | pornographic material depicting minor* |
| 14 | technology facilitated child sexual abuse |
| 15 | technology mediated child sexual abuse |
| 16 | technology assisted child sexual abuse |
| 17 | 1 or 2 or 3 or 4 or 5 or 6 or 7 or 8 or 9 or 10 or 11 or 12 or 13 or 14 or 15 or 16 |
| 18 | limit to english language |
